# An Image-Based Data-Driven Model for Texture Inspection of Ground Workpieces

**DOI:** 10.3390/s22145192

**Published:** 2022-07-11

**Authors:** Yu-Hsun Wang, Jing-Yu Lai, Yuan-Chieh Lo, Chih-Hsuan Shih, Pei-Chun Lin

**Affiliations:** 1 Department of Mechanical Engineering, National Taiwan University, Taipei 10617, Taiwan; r06522806@ntu.edu.tw (Y.-H.W.); r09522801@ntu.edu.tw (J.-Y.L.); 2 Mechanical and Mechatronics System Research Laboratories, Industrial Technology Research Institute, Hsinchu 31040, Taiwan; yuanchiehlo@itri.org.tw (Y.-C.L.); itria20303@itri.org.tw (C.-H.S.)

**Keywords:** convolution neural networks, transfer learning, grinding, abrasive belt, surface images, surface roughness, grit number

## Abstract

Nowadays, the grinding process is mostly automatic, yet post-grinding quality inspection is mostly carried out manually. Although the conventional inspection technique may have cumbersome setup and tuning processes, the data-driven model, with its vision-based dataset, provides an opportunity to automate the inspection process. In this study, a convolutional neural network technique with transfer learning is proposed for three kinds of inspections based on 750–1000 surface raw images of the ground workpieces in each task: classifying the grit number of the abrasive belt that grinds the workpiece, estimating the surface roughness of the ground workpiece, and classifying the degree of wear of the abrasive belts. The results show that a deep convolutional neural network can recognize the texture on the abrasive surface images and that the classification model can achieve an accuracy of 0.9 or higher. In addition, the external coaxial white light was the most suitable light source among the three tested light sources: the external coaxial white light, the high-angle ring light, and the external coaxial red light. Finally, the model that classifies the degree of wear of the abrasive belts can also be utilized as the abrasive belt life estimator.

## 1. Introduction

Belt grinding is a commonly used finishing process that can reduce the defects and burrs created by previous machining procedures. Because of the flexible characteristics of the contact surface of manufacturing machines, belt grinding is more suitable for machining workpieces with complicated surfaces, such as turbine blades and faucets. Although much research on the machining process has been reported, inspections after grinding are less discussed. Manish et al. [1] established a visual inspection system using a histogram of the pixel intensity and Canny edge detection to detect poorly ground areas. Caesarendra et al. [2] created a convolutional neural network (CNN) model to estimate grinding tool wear. By evaluating the inputs of vibration and force measured and their signal combinations during the manufacturing process as inputs, an optimal CNN model was produced. Pandiyan et al. [3] explored how manufacturing parameters such as cutting speed, force, polymer wheel hardness, feed, and grit size of abrasive belts affected the material removal rate of a belt grinding process. By applying six different regression methodologies to the experiment data, the authors concluded that the material removal rate was prone to be nonlinear to the grinding parameters, meaning that nonlinear models are more suitable. In addition, all studies have shown that the grit size of an abrasive belt is the most significant factor in the material removal rate. Bi et al. [4] presented a convolutional neural network with an original time waveform of acoustic emission (AE) signals as the input built for multiclass classification of wheel states. The paper also presented a long–short-term memory (LSTM) model to predict wheel states based on frequency domain AE signals. Wang et al. [5] designed a stacked autoencoder and long–short-term memory network (SAE–LSTM) using tool wear conditions and sensor signals as the inputs to predict surface roughness during CNC machining. Transfer learning was applied to train the prediction model, and machining experiments for the assembly interface using Ti6Al4V as an aircraft’s vertical tail material were conducted.

Although few studies have addressed visual inspection for ground workpieces, visual inspection for other applications can be found. For example, Haralick et al. [6] proposed a gray-scale co-occurrence matrix to extract topographic image features, achieving an accuracy of about 80%. Tsai et al. [7] used a co-occurrence matrix to extract image features as six parameters, which were then input into a neural network for classification to detect the defects on fabric surfaces. Xie [8] collated the previous approaches for defect inspection using machine vision, classifying them into four major approaches: statistical, structural, filter-based, and model-based. Tian et al. [9] developed machine vision with different lighting modes to segment the region of interest and detect defects. The paper showed that the grinding mark angle is highly correlated with the average gray level of the image, so in the experiment, they used multidirectional light sources to indicate the different grinding mark directions of the defects. The method can recognize five kinds of defects on ground flat parts, and the experiments show that the accuracy of the inspection system can approach 100%. Luo et al. [10] proposed a defect detection method based on image data. First, a median filter was applied to images to eliminate noise; then, the Sobel operator was applied to the image with eight gradients in the different directions. Finally, the thresholds were used to categorize the defective areas.

The recent development of machine learning techniques strongly helps develop visual inspection. Czimmermann et al. [11] reviewed the visual inspection methods commonly used in industry, concluding that deep learning can be used to perform most visual classification tasks. Yet the size and diversity of the training dataset, as well as the computation resources, need to be traded for the model’s generalizability and accuracy. Suen et al. [12] developed a dataset of images corresponding to surface roughness after milling and used this dataset to train a convolutional neural network to classify the surface roughness level of the milling surface by images. The images were first processed by Hough transform for skew correction, improved Sobel operator for edge highlighting, and complex wavelet transform for image feature enhancement before being input into a neural network, which was modeled after the ResNet-styled infrastructure [13] for supervised learning. The neural network was not the classical ResNet neural network structure, so the weights of the neural network had to be trained from scratch. Arriandiaga et al. [14] created a recurrent neural network-based virtual sensor to estimate the wheel wear and surface roughness of the grinding process. The sensor used grinding parameters and the power consumption of the wheel spindle to predict the targets. It showed that during the fine-tuning process, the mean square error (MSE) may not be sufficient for showing the performance of the model; thus, two indicators are proposed: AME and saturation. From the results, Arriandiaga inferred that the grinding energy has a greater effect on the surface roughness modeling than on wheel wear modeling. Yang et al. [15] proposed a real-time, small-part defect detection system for manufacturing that was based on the defects of a 0.8 cm darning needle. The authors proposed a defect detection algorithm for tiny parts that were based on a single short detector network (SSD) and deep learning. He et al. [16] presented a defect detection network (DDN) for steel plates and set up a defect detection image data set—NEU-DET—for training and evaluating model performance.

In the paper, a deep learning model structure was proposed to obtain the specific category and detailed location of a defect by fusing the multilevel features. Tao et al. [17] showed a novel CNN-based architecture that could be utilized in both defect detection and classification tasks for a metallic surface against complex industrial scenarios. In the paper, a novel cascaded autoencoder (CASAE) architecture was designed for segmenting and localizing defects. Then, a compact convolutional neural network (CNN) was used to classify the defect regions of the segmented results into their specific classes. Wieczorek et al. [18] developed a CNN model to classify the images of three different drilled states. The data underwent a series of pre-processing procedures, including normalized, scaled-down, and additional instances that were created with the use of data-augmentation techniques, a self-developed transformation, and general adversarial networks. When trained on the same data, the prediction result was found to be better than Microsoft’s Custom Vision service.

Transfer learning can further help improve deep learning methods on image-related tasks. Pan et al. [19] reviewed the literature on transfer learning for traditional machine learning, defining transfer learning in detail. A conceptual classification of different transfer learning goals was made based on the relative relationship between the source domain and target domain and the source and target task. Tan et al. [20] reviewed the literature on deep learning approaches for transfer learning into four major categories—instance-based, mapping-based, network-based, and adversarial-based—and also defined various migratory learning approaches. Currently, CNNs for visual image recognition are the most suitable for network-based learning, that is, by using the neural networks and weight parameters that have been trained in advance with other datasets and then performing further training on the target task. Oquab et al. [21] used the network-based transfer learning described above, using the structure and parameters of the first few layers of AlexNet [22], which had been pre-trained in the ImageNet dataset, and adding additional fully connected layers with data labels in the target domain. Finally, a neural network capable of object detection and motion classification was trained, and an average accuracy of 70% was achieved on the motion classification task of Pascal VOC 2012. However, because of the requirement of deep learning to have a comprehensive dataset, Ackay et al. [23] proposed a generative adversarial network (GAN) to automatically generate new data for training. Tang et al. [24] further developed a dual auto-encoder generative adversarial network (DAGAN) to improve the accuracy of neural network recognition and speed up training convergence based on the same GAN-based strategy. This was applied to the publicly available industrial inspection training dataset MVTec AD, which contains the cell phone screen and wood defects datasets. The results show that the model can be trained with 80% accuracy without a large number of images.

Motivated by the recently well-developed data-driven techniques and the prevalence of vision-based sensing techniques in factory automation, the current study aims to develop an image-based method capable of detecting the surface texture quality of a grounded workpiece on three different aspects: grit numbers of abrasive belts, surface roughness, and degree of wear of abrasive belts. The quality of a grounded surface is usually judged by the surface roughness, which is a geometrical characteristic and usually relies on precision optical measurement or contact measurement. The present work explores whether geometrical surface properties, such as roughness, can be captured by imaged texture. More specifically, the current study investigates whether geometrical characteristics can be extracted by color characteristics. The first task can be regarded as the first step of the second task because it links the geometrical property to the imaged texture. The degree of wear of an abrasive belt can also be regarded as a geometrical property change, and it greatly determines grinding performance. Thus, estimating the degree of wear is the third task, which utilizes a similar technique. The data-driven models of the three tasks utilize transfer learning based on other pre-trained CNNs. The three tasks were experimental and individually evaluated to individually validate the performance of the model. The results show that the proposed strategy enables the CNN models to quickly fulfill the classification and regression tasks, confirming that the CNN can distinguish the fine textures of differently grounded workpieces.

The current research provides a few contributions. The proposed method utilizes only a small dataset to train the model, which can distinguish the mesh number of the abrasive belt used to grind the workpieces, hence predicting the surface roughness of a local region on a workpiece and classifying the degree of wear of the abrasive belt. The benefit of transfer learning is that it allows this method to train a model with a small dataset. Compared with other studies, Sun et al. [12] used 3700 records of images as a dataset to train a ResNet-based CNN from scratch, and the dataset must go through image pre-processing as well. Most of the studies [25,26] on the degree of wear of abrasive belts have mainly focused on the conditions of the abrasive belt or the grinding acoustic signal of the abrasive belts [27,28], while the present research concentrates on the surface texture of the workpieces after grinding. As summarized by Xie et al. [8] and reported in the introduction of the manuscript, most defect detection techniques focus on local defects, not on tonality defects. Owing to a different target application, there is no direct comparison between their works and the current research. For tasks 1 and 2, only 40 raw images taken from 20 workpieces were utilized to train the neural networks, and 30 raw images taken from 15 workpieces were used in task 3.

The possibility of adapting other methods to the tasks performed in the current research has been investigated. However, owing to differences in the applications, we found it difficult to adopt other methods directly. For example, the work reported by Suen et al., is similar to ours, where surface roughness is estimated using images. In their case, because the surface after milling has a more distinct and sharper pattern, the data would require pre-processing before training. By contrast, the surface after grinding has subtle and random patterns, so no pre-processing is required. The work reported by Tian et al., is related to the ground surface as well, but their work focuses on finding a method for distinguishing the anomaly pattern from the normal pattern based on multidirectional light sources. This is different from the current research, which focuses on using surface images to estimate the roughness or degree of wear of the abrasive belt. In short, because of the differences resulting from the applications themselves, it is difficult to deploy other methods directly in this case for a fair comparison. This situation is unlike the usual case, where developers try to find the best model using the same dataset.

The remainder of the current paper is organized as follows: Section 2 describes the experimental setup, the image data collecting process, and the data-driven models utilized in this work. Section 3 reports the evaluation results and discusses the results. Section 4 concludes the work.

## 2. Data Preparation and Models

### 2.1. Data Preparation

**Figure 1:**
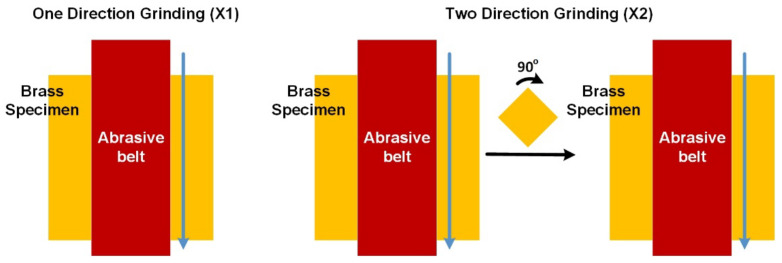
Two kinds of grinding processes utilized in this work: one-direction grinding and two-orthogonal-direction grinding.

The workpiece preparation is as follows: The workpiece was made of brass (i.e., the same material as general water faucets) with dimensions of 50 mm in length and width and 2 mm in thickness. The workpieces were manually ground on a belt sander with abrasive belts of five different grits: #100, #150, #240, #400, and #600. Although manually performed, the grinding process was standardized, and the difference in the amount of material removed was within a reasonable range. The specimens were ground in either one direction (X1) or in two orthogonal directions (X2), as shown in Figure 1. In addition, two workpieces were prepared for each grit number and ground direction. As a result, there were 20 workpieces, as shown in Table 1.

**Figure 2:**
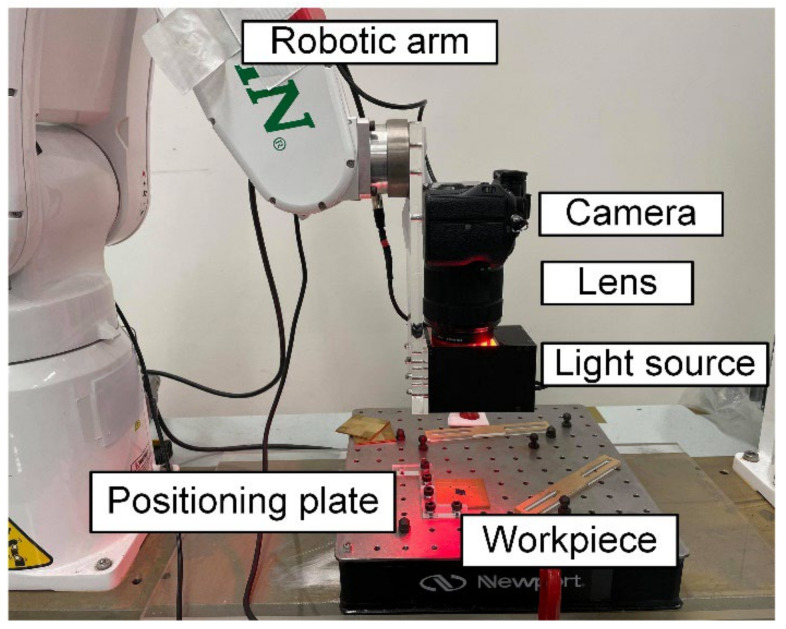
The setup of images acquisition system to take photos of the ground workpieces.

**Figure 3:**
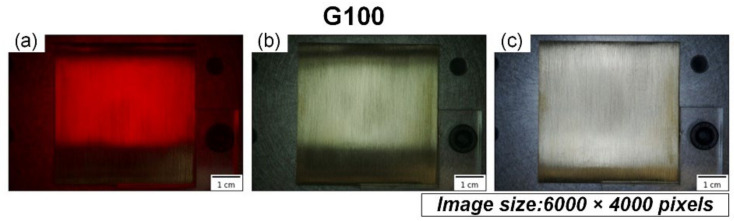
The raw images of the same workpiece illuminated by three different light sources: (**a**) external coaxial red light; (**b**) external coaxial white light; (**c**) high-angle ring white light.

The images of the workpieces were taken using the setup shown in Figure 2. The camera (A7 III, SONY) was positioned vertically to capture the surface of the workpiece, which was placed horizontally. Each of the 20 workpieces was photographed using three light sources: external coaxial red light, external coaxial white light, and high-angle ring light, as shown in Figure 3. For each light source, the workpiece was photographed twice, with different orientations positioned in an orthogonal manner to each other. The images of the ground workpieces using different light sources belong to different datasets; these were fed to the model and trained separately so that the training results of the model could be utilized to suggest a suitable light source for texture detection.

**Figure 4:**
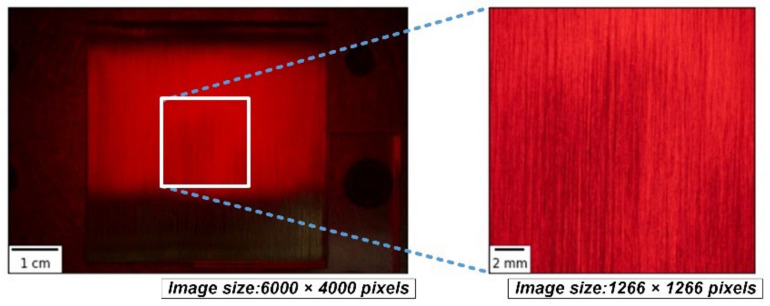
The first-step crop of the raw image of the workpiece.

**Figure 5:**
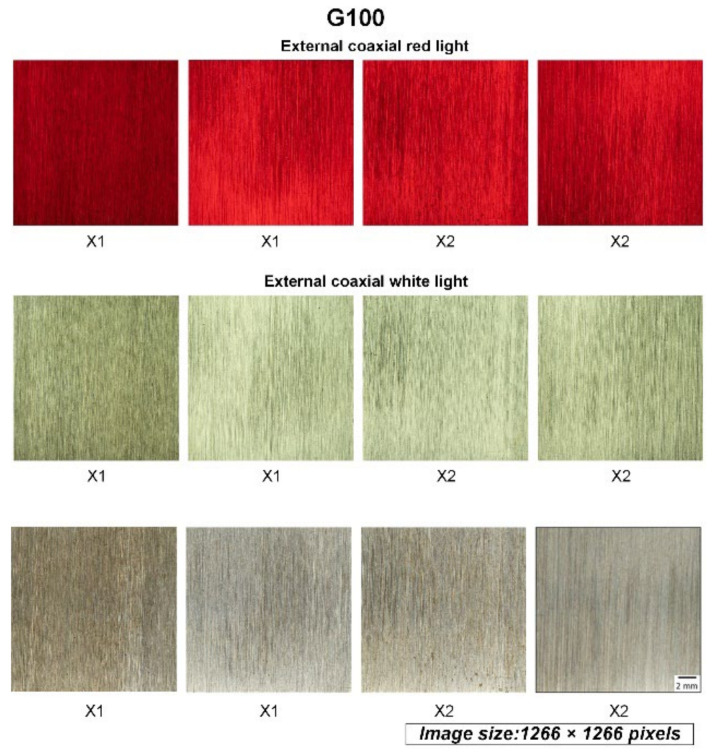
The images of the workpiece after the first-step cropping of the raw images. X1 and X2 represent one-direction grinding and two-orthogonal-direction grinding, respectively.

**Figure 6:**
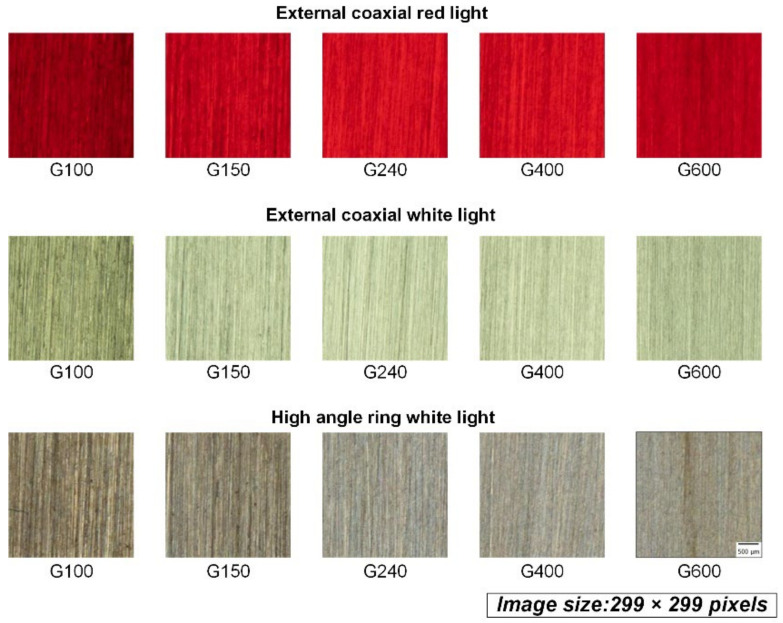
The images of the workpieces after second-step cropping.

Two-step cropping of the raw images was utilized to prepare the final images for the model. First-step cropping was performed to crop the center 1266 × 1266 pixels of the raw image of the workpiece, which was uniformly ground, here corresponding to the area of 18 × 18 mm in the center of the workpiece, as shown in Figure 4. Figure 5 shows the exemplary cropped images of the workpiece taken by three different light sources. All images in the figure were processed by abrasive belt grinding with grit #100 (i.e., label G100). The second-step cropping was performed to crop the images to a suitable pixel size for various network models because 1266 × 1266 pixels is still too large for networks, and because each model uses a different pixel size. Cropping is better than downsizing because the latter may result in a loss of surface texture details. Figure 6 shows an exemplary set where the images are 299 × 299 pixels in size. In Figure 6, the workpieces ground by different grits are shown. After two-step cropping, there were 1000 images in total and 200 images for each grit. Note that all the images were taken from different regions of the same workpiece or different workpieces so that they can be regarded as “raw” images for the model.

**Figure 7:**
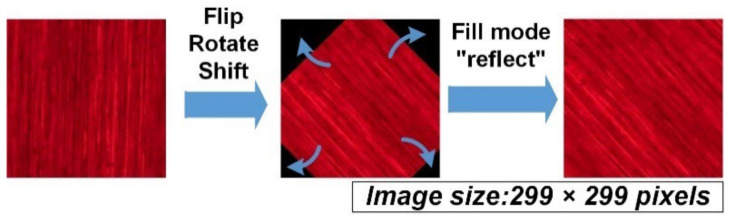
Data augmentation using rotation and flip.

The images were augmented before being imported into the model for training. One of the reasons for doing this was to increase the amount of image data. The other was to increase the model’s capability of classification. The raw images were taken at a fixed configuration, where the surface texture was aligned in a certain direction. In empirical applications, the texture may be randomly distributed. Thus, in augmentation, the raw images were randomly rotated within a range of 180 degrees and flipped along the vertical and horizontal axis. After rotation, the missing corners were filled by mirroring the image inside the crop, as shown in Figure 7. Scaling was not utilized because it may alter the texture size, which is related to belt grit. Data augmentation was implemented using Keras [29].

**Figure 8:**
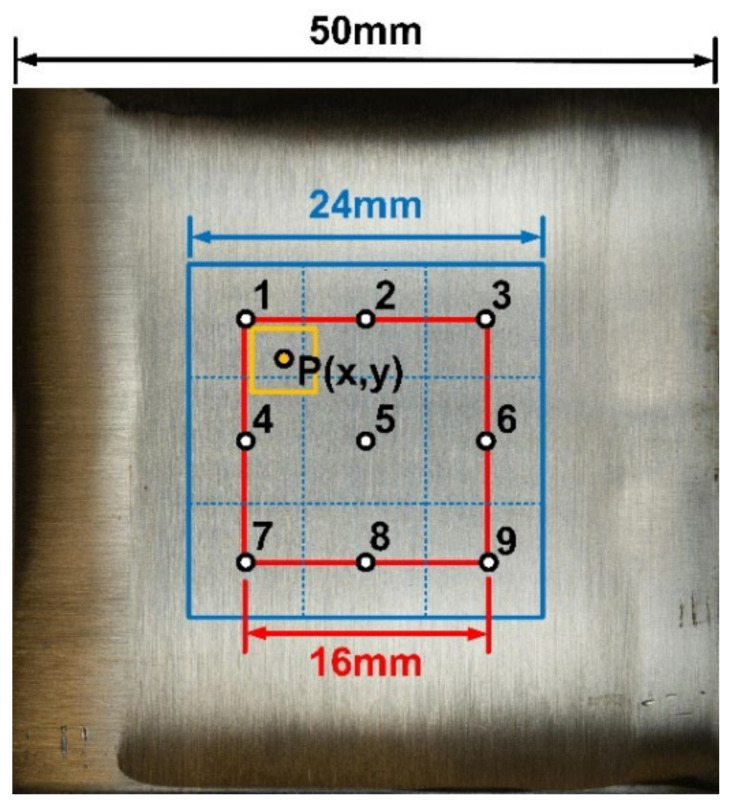
The notations and definitions of the workpiece utilized for measuring and computing the surface roughness of the workpiece.

**Figure 9:**
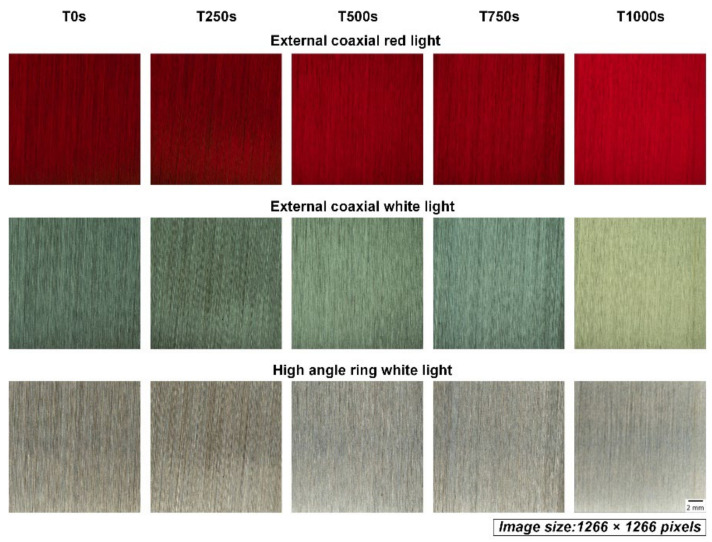
The images of the workpieces ground by abrasive belts with different degrees of wear.

The work included three different detection tasks, so the labeling of these three tasks was carried out in different fashions. The first task was to estimate the grit number of the abrasive belt using the surface image of the workpiece. Thus, the actual grit number was the label for the image data. In the experiments, the abrasive belts of six git numbers were utilized, including #100, #150, #240, #400, and #600. Thus, the labels G100, G150, G240, G400, and G600 were simply defined. The second task was to estimate the surface roughness of the workpieces using the surface image of the workpiece. Thus, the surface roughness was the label requiring empirical measurement to yield the ground truth. The surface of the workpiece with a squared area of 24 × 24 mm was divided into nine subsquares (i.e., blue boxes), and the surface roughness of each subsquare was measured using the surface roughness tester (SJ-210, Mitutoyo, Tokyo, Japan), as shown in Figure 8. Three measurements were taken, and their average was set as the roughness at this specific point. Here, the arithmetic average roughness (Ra) was used as the indicator. The images for labeling were the cropped images (i.e., yellow boxes) of the square formed by nine measured points (i.e., red box). The roughness representing the yellow-cropped images needed to be recomputed; hence, the roughness of the center point P of each yellow box was computed using bilateral interpolation, and this roughness represented the roughness of this specific box. The third task was to estimate the degree of wear of the abrasive belt. Thus, the labels of the images were the accumulated pre-grinding time of the abrasive belts, as shown in Figure 9. The roughness measurements of this dataset were identical to the set utilized in the second task.

### 2.2. Preparing Abrasive Belts with Different Degrees of Wearing

Tool life estimation has always been challenging in real-life manufacturing processes [30]. Frequent tool changes can lead to an increase in the cost of consumables; however, there are random and sudden stoppages when tool life is limited or the surface quality of a batch is not found to be up to standard during quality inspection. The same problem also occurs in the grinding process. The material removal rate of the abrasive belt decreases with the grinding process, and the actual manufacturing process repeatedly uses the same trajectory for each workpiece. When the material removal rate decreases, there will be an underground area, increasing the defective rate. However, after adjusting the processing parameters (e.g., belt speed, grinding force, and grinding track), it is necessary to go through several machining and manual inspections to obtain the empirical value of belt replacement, which will take a lot of time and create many defective products.

**Figure 10:**
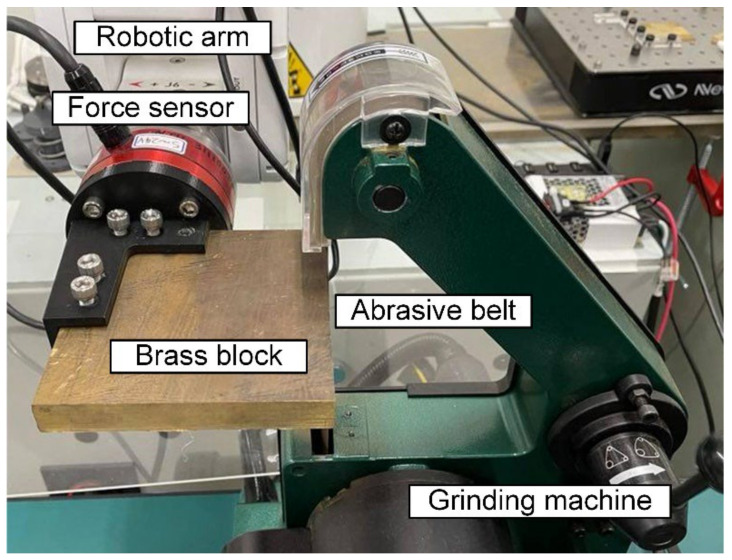
The setup to systematically prepare the abrasive belts with different degrees of wear.

To perform the third task with consistent and uniform characteristics, belts with different degrees of wear should be systematically generated. Thus, an experimental setup that wears the abrasive belt with a fixed grinding normal force was constructed, as shown in Figure 10. A brass block was used as the grinding workpiece, and the normal grinding force was measured using a force sensor; the force was sent back to the arm for a PID constant force control of 5 N. The operation variable was the grinding time. In addition to preparing abrasive belts with different degrees of wear, the abrasive belt wear time had the same time interval to better quantify the degree of wear. The experiments used a belt with grit number #100. The contact area between the abrasive belt and the brass block was 381 mm^2^. The cumulative grinding times were 0, 250, 500, 750, and 1000 s of grinding. The interval of 250 s was obtained using the actual grinding information, which considers the actual belt length and speed, the number of seconds of grinding per machining track, and the number of workpieces machined before the belt is replaced, as shown in Table 2.

It was assumed that the two factors most relevant to the degree of wear of an abrasive belt are “grinding normal pressure” and “total grinding contact length at a single point on the belt.” The above table shows that the normal grinding pressure in the experiment is about twice that in the factory because the belt sander used in the experiment was small, and if the normal force is assumed to be 10 N, there will be a high heat problem. In addition, the deformation of the belt caused by the 10 N grinding normal force will make the material removal rate on the surface of the workpiece uneven. In Table 2, because there is only a single block of the workpiece in the experiment, it is necessary to consider how to design the length of the grinding contact time so that the degree of wear of the abrasive belt can be comparable with the actual machining process. Disregarding the influence of the different degrees of wear caused by normal grinding pressure, let the linear speed of the abrasive belt be *V*, the processing time of a single workpiece on the same abrasive belt *t*, the circumference of the abrasive belt *C*, the number of workpieces processed before the abrasive belt is retired *N*, and the length of a single abrasion *l*. The following equation can be used to calculate the total grinding length *L* of a point on the abrasive belt:

(1) $$\frac{V\;\left[\frac{mm}{s}\right]\times t\;\left[s\right]}{C\;\left[mm\right]}\times N\times l\;\left[mm\right]=L\;\left[mm\right]$$

To make the wearing conditions of the factory process equal to the total grinding contact length *L* in the accelerated test, the following equation can be listed using, where the subscript *m* is used for the factory parameters and *a* for the accelerated test:

(2) $$\frac{V_{m}\times t_{m}}{C_{m}}\times N_{m}\times l_{m}=\frac{V_{a}\times t_{a}}{C_{a}}\times N_{a}\times l_{a}$$

As described earlier, this experiment replaces the new workpiece in the factory process with continuous force-controlled grinding, so $t_{a}$ and $N_{a}$ are not available, which means that both are replaced by the desired parameter—the total grinding contact time $T_{a}\;$—as follows:

(3) $$\frac{V_{m}\times t_{m}}{C_{m}}\times N_{m}\times l_{m}=\frac{V_{a}}{C_{a}}\times T_{a}\times l_{a}$$

After substituting the parameters according to Table 2, the following equation can be obtained:

(4) $$\frac{16,338\times 25}{3500}\times 150\times 50=\frac{16,000}{762}\times T_{a}\times 15\;\Rightarrow T_{a}\approx 2779\;s$$

The total grinding contact time calculated above is about 2779 s, which translates to about 46 min, which means it would take a lot of time to set 500 s as the time interval for the total processing contact time. Therefore, this experiment set the time interval to 250 s, producing five different degrees of wear of abrasive belts, and the images of the workpieces ground with these belts were then labeled with the corresponding data: T0s, T250s, T500s, T750s, and T1000s.

### 2.3. Data-Driven Models

Instead of finding a new architecture, this work investigates a suitable architecture for industrial applications where a small amount of data is available. This is challenging yet useful for deploying the methodology in real factory automation processes.

**Figure 11:**
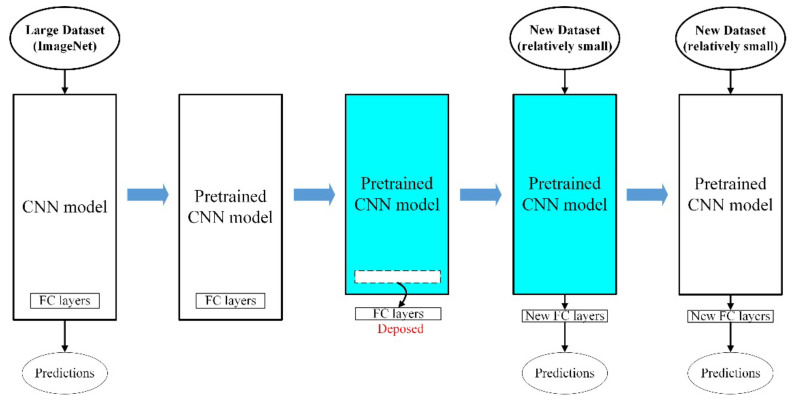
The diagram of the transfer learning steps.

The current work utilized transfer learning to reduce the size of the dataset to train the convolution neural networks, as well as to decrease the computation time for training. Each workpiece needs to be cut and ground and then either captured by a camera or measured using a surface roughness tester, which is extremely time consuming. Using a model that has been pre-trained on a large number of datasets and then applied to the small dataset under study can lead to fast and reliable results. This experiment was implemented according to the official Keras instructions [31], as visualized in Figure 11. It shows the transfer learning process adopted in our work. The parameters were trained twice using different learning rates. The pseudocode is listed as Algorithm 1.
**Algorithm 1** Pseudo Code of the Model Training Process**IMPORT** libraries, including Tensorflow Keras, Scikit Learn. **SET** training parameters. **SET** the training and validation image data generator, respectively, to provide the model’s pre-processed images. **SET** the machine learning model structure. In the first stage, the parameters of the base model should be frozen. **SET** the loss, optimizer, and metrics for the model. **FIT** the model for training the added layers. **SET** the parameters of the base model to be trainable. **SET** the learning rate to a much smaller value. **FIT** the model for fine-tuning.

**Figure 12:**
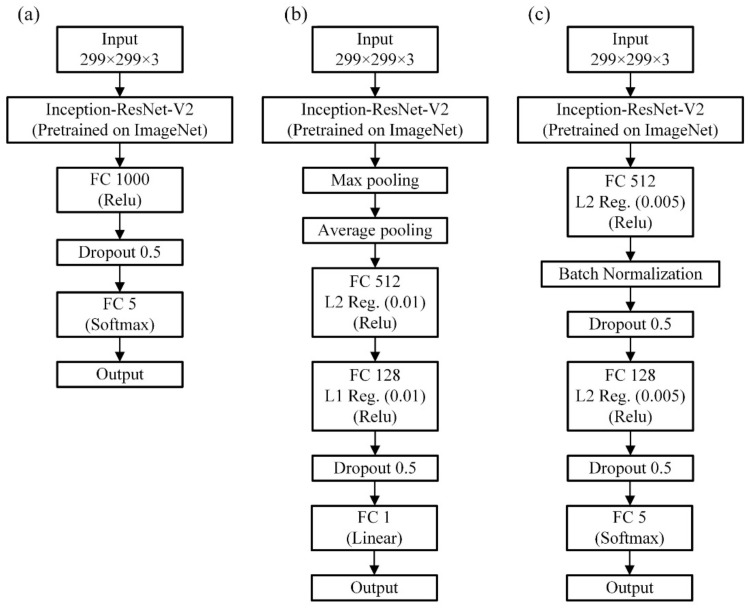
The classification model (**a**), the regression model (**b**), and the classification model (**c**) are utilized for the first, second, and third tasks, respectively.

The first task was to estimate the grit number of the abrasive belts that grind the workpiece, which can be considered a classification task. The performance of several pre-trained convolutional neural network models was evaluated, and the best one was utilized for the second and third tasks. For the first models, the pre-trained structure was followed by a fully-connected layer with 1000 neurons, a dropout layer, and then output to the SoftMax function of each class, as shown in Figure 12a. The dataset has 1000 images in total and 200 images for each grit, as shown in Figure 6.

The second task was to estimate the surface roughness of the grounded workpiece. The model structure was very similar to that of the first task, and the main difference was the new fully connected layer (FC layer) structure. The final output layer of the first task was the category number of neurons, and the output value was obtained by the SoftMax function. In contrast, the second task was a regression task, so the final output layer had only one neuron, with the final value being output by the linear function, as shown in Figure 12b. Here, the loss function also changed from categorical cross-entropy to MSE. Experience indicates that it is better to design new FC layers in the training process in the depth direction for better performance. The pooling layers were used to prevent the overfitting of the model by properly decreasing some local information in the feature maps. Similar to the first task, the dataset has 1000 images in total and 200 images for each grit, as shown in Figure 6.

The third task was to estimate different degrees of wearing of the abrasive belts, which was the classification task, so the model structure, as shown in Figure 12c, was similar to the first task. The deeper the network, the more complex the network needs to be so that a more nonlinear model can be trained with fewer parameters [27]. The first task was simply to distinguish the images from the different numbers of abrasive belts. The difference in abrasive particles between the different numbers can be quite large, which makes it easier to distinguish grinding patterns. However, the third task was the same number of abrasive belts with changes in time and reduced the grinding capacity; grinding out the pattern is more difficult to distinguish. Because the model was deeper, the dropout layer, regularization of the weights, or batch normalization layer was implemented to decrease the possible overfitting problem. Using pooling before reducing the two-dimensional feature maps to one-dimensional tensors not only accelerates the convergence but also brings some regularization effect. The dataset has 750 images in total and 150 images for each cumulative grinding time, as shown in Figure 9.

The training was executed on the platform with a RAM of 25 GB and the Nvidia Tesla V100 SXM2 16 GB GPU offered by Google Colaboratory. During training, around 9 GB of GPU memory was used.

## 3. Results and Discussion

### 3.1. The First Task: Classification of the Grit Number of the Abrasive Belts

The first experiment compared the performance of different CNN models using the external coaxial red light dataset to select the best base model for subsequent experiments. Four different models were evaluated, including ResNet V50, ResNet V152, Inception V3, and Inception-ResNet V2, as shown in Table 3. Each model was evaluated three times, and the averaged results are presented. In addition, the results after the initial training and fine-tuning training are listed. Table 3 also records the suitable dropout ratios for each model. The dropout layer was used to slow down the overfitting of the models, and the comparison adjusted different dropout ratios for different models to achieve the best score for each model under this structure. Table 3 reveals that the model’s accuracy after the first training was around 0.7 to 0.8. The parameters of the pre-trained model from transfer learning were frozen during the first training. The dataset for the pre-trained model—the ImageNet dataset—had different features from the dataset of this work, so the accuracy was not as high as expected. The parameters of the convolutional layer were updated in the fine-tuning stage, so the accuracy improved to around 0.9. The best model utilized the Inception-ResNet-V2 model as the base model. Traditional machine vision requires light sources close to the color of the subject material, so red light is the most suitable for brass specimens. Therefore, in the first set of experiments, the best neural network structure was selected based on the dataset using red light. Later, the follow-up experiments revealed that the best model was the one trained with the white coaxial light dataset, as shown in Table 4.

**Figure 13:**
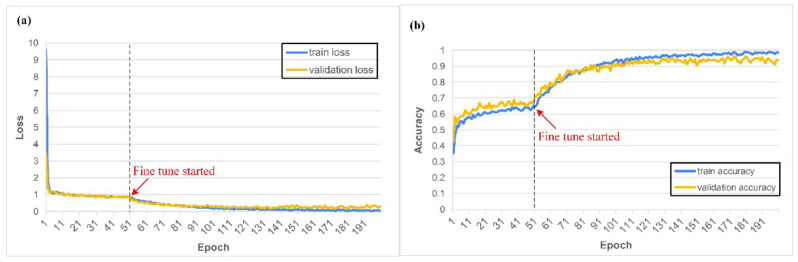
The loss (**a**) and accuracy (**b**) of the model of the first task during the learning process.

**Figure 14:**
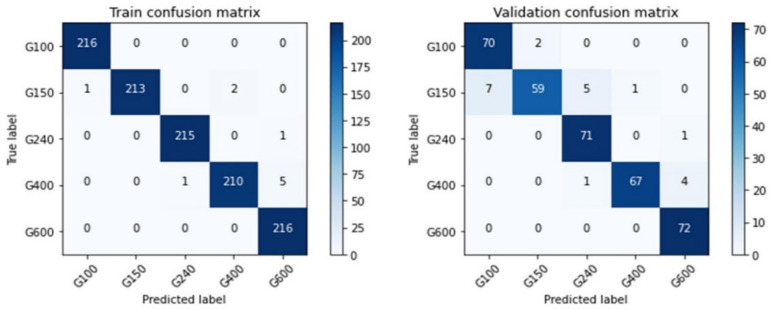
The confusion matrices of the model of the first task.

Following the results of the first experiment, the Inception-ResNet-V2 model was trained using the external coaxial white light dataset; the parameters are shown in Table 5. Task 1 takes about 1 h to train 200 epochs. During training, it was found that the Adam algorithm used for updating the weights of the neural network had the advantage of fast convergence. It also exhibited a more pronounced spike than the other algorithms when the mini-batch contained special data [32]. After testing, it was found that this situation can be more moderate when the batch size is 32 than 16 [33]. The learning process after training is shown in Figure 13, where the black dashed line separates the first training and fine-tuning processes. Figure 13 shows the relationship between loss and accuracy during training, and the curve shape reveals the training status. Figure 13b reveals that the accuracy after the first training was about 0.6 to 0.7, and that after this, the fine-tuning process can increase to more than 0.9. Although the data of this task were very different from that of ImageNet, the designed model structure and pre-trained weight directions still provided a good starting point for the model to converge in the right direction. When the training continued, the model started to overfit, and here, the model performed better on the training set than on the validation set. At this point, the dropout ratio made the training loss as close to the validation loss as possible without losing too much validation accuracy. Figure 14 shows the confusion matrix of the training results. The Inception-ResNet-V2-based model was the most accurate in determining the grit number of the #240 abrasive belt, followed by #100 and #600, and less accurate in distinguishing the grinding marks of the #150 and #400 belts. Figure 14 is utilized to understand the model’s performance on different types of workpieces. To clarify the presentation, we have rewritten the contents related to these three figures.

As the final comparison of the first task, Table 5 shows the model’s accuracy fed by the three datasets using different light sources. When training the models separately, the model structure and tuning process were identical. Table 5 shows that the dataset with an external coaxial white light had the best performance, followed by the high-angle ring white light, and finally the external coaxial red light. The basic convolutional neural model, Inception-ResNet-V2, used a large dataset (ImageNet) with color data for pretraining, and the model’s input was designed for RGB tricolor data. When using red light, the entire image is almost entirely red in hue, meaning that the other two-color lights, blue and green, are quite low in brightness, thus losing some of the features that help identify the image. The performance of the dataset of the external coaxial light source was better than that of the high-angle ring light source, probably because the illumination effect of the high-angle ring light is not the same in different areas of the same test piece. As shown in Figure 5, the brightness of the outer coaxial white light image is more uniform than that of the high-angle annular white light image, hence allowing features to be detected more evenly throughout the image. As a result, in the following experiments, the dataset using external coaxial white light was utilized.

### 3.2. The Second Task: Estimation of the Surface Roughness of the Workpiece

**Figure 15:**
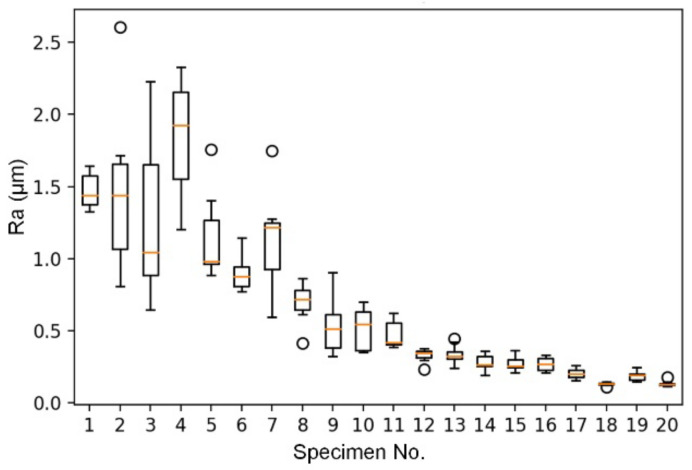
The box-and-whisker plot of the surface roughness Ra of the workpieces.

Figure 15 shows the box-and-whisker plot of the nine measurements for each workpiece, and in total, 20 workpieces were indexed in the horizontal axis and listed in Table 1. The Ra of the workpiece ground by coarse abrasive belts was generally greater than that of fine abrasive belts, as expected, and the variation of the Ra had a similar trend as well. The variation of the Ra of the workpieces ground by the #100 abrasive belt (i.e., workpieces 1~4) was greater in the workpieces with two-orthogonal-direction grinding (i.e., workpieces 3 and 4) than in the workpieces with one-direction grinding (i.e., workpieces 1 and 2). We suspect the vibration caused by the abrasive belt with coarser particles was greater, making the grinding surface more prone to uneven grinding. The second grinding in the orthogonal direction further aggravated this situation. This phenomenon will tend to decrease as the size of the abrasive grit becomes smaller. Because the abrasive friction and vibration of the fine grit abrasive belt are smaller, the process is more stable, and the uniform grinding effect of the second grinding can be seen at this time. The error in this experiment may also be because of the manual grinding of the workpieces instead of using a robot arm to pick up the workpieces and walk them on a fixed track. The grinding process was based on the machining operator’s experience to determine the uniformity of the grinding results, and the variation of the measured values may be larger. Though the experimental results reveal that the relation between the surface roughness and different grit numbers may not be correlated perfectly, the grit number of the abrasive belts did have a significant correlation with the ground surface roughness value. Thus, the data-driven model can be deployed further.

**Figure 16:**
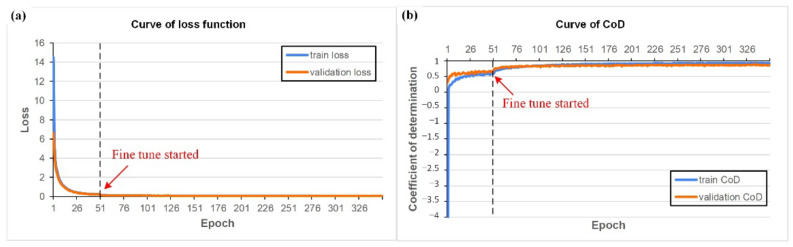
The loss (**a**) and coefficient of determination (**b**) of the model for the second task.

**Figure 17:**
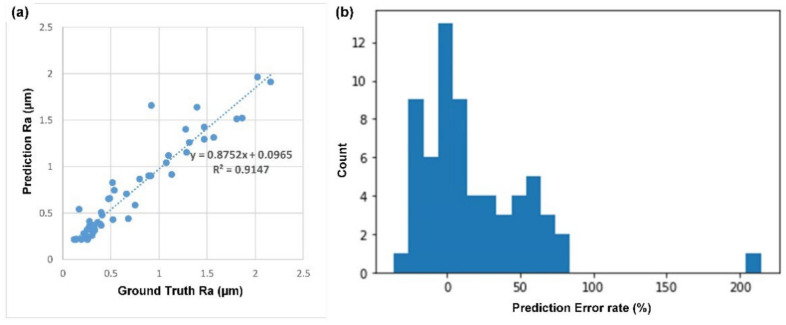
Performance of the second task: Estimation of the surface roughness Ra: (**a**) Scatter diagram of the estimated Ra versus the ground truth and (**b**) histogram of the Ra error rate.

Based on the results showing that the CNN model can classify the grit number of the abrasive belt and that the grit number of the abrasive belt was correlated with the surface roughness, the goal of the second task was to estimate the surface roughness using the same image source. Table 6 shows the parameters used in the training process, and Figure 16 shows the curves of the learning process. Task 2 takes about 2 h to train 350 epochs. In Figure 16b, the coefficient of determination (CoD) was utilized to evaluate the model’s performance for the second task because it was a regression task, not a classification task. If the same range of the numerical error in the ground truth value is small or large, the calculated error rate would be much different. If we were to mix them directly, it would not be easy to see an improvement in the results. For the regression task, we chose to look at the CoD. Figure 17a shows that the CNN model can estimate the Ra value to a considerable degree, and the CoD between the estimated value and actual value can reach 0.8 to 0.9. Figure 17b shows that for the workpieces with roughness ranging from 0.2~2.2 um, about 75% of the 64 samples in the evaluation can be kept within 50% of the error range.

The current work utilized a planar workpiece, and the best direction for visual inspection, in this case, would be the normal direction of the plane. This assumption originated from conventional machine vision. In contrast, experienced human inspectors are prone to slightly rotate the workpiece for inspection, so it is also possible that different directions of the surface images can offer different information about the surface roughness.

### 3.3. The Third Task: Estimation of Different Degrees of Wear of the Abrasive Belts

**Figure 18:**
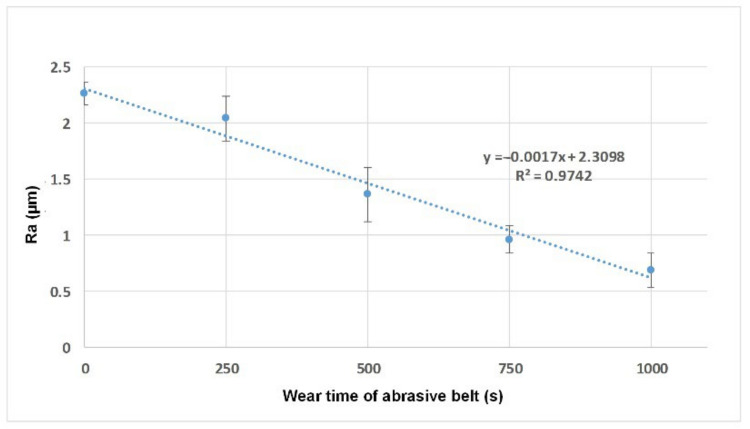
The scatter diagram of the measured Ra versus the abrasive belt with different degrees of wear.

The first and second tasks revealed that the CNN model with vision-based data could distinguish the geometrical and abrasive patterns on the surface of the workpiece. Thus, as the “inverse” problem, the third task aimed to estimate the degree of wear of the abrasive belt. As described in Section 2.2, five degrees of wear of the abrasive belts were prepared using an acceleration test. Together with the surface images of the workpieces, the dataset was formed for training. Before implementation, whether a certain relation existed between the surface roughness and abrasive belt with different degrees of wear should be evaluated first. The results are shown in Figure 18, which indicates that the Ra of the workpiece is highly linearly correlated to the abrasive belt with different degrees of wear. Following the second task, where the images can be used to identify the surface roughness, the estimation can be further extended to the condition of the abrasive belt.

**Figure 19:**
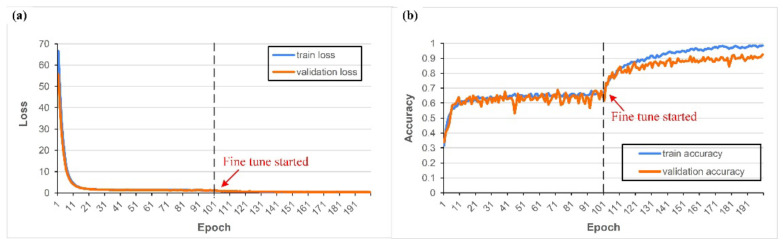
The loss (**a**) and accuracy (**b**) of the model of the third task.

Table 7 lists the training parameters, and Figure 19 shows the learning process curves. With the dataset with external coaxial white light as the light source, the model achieved 90% accuracy. Task 3 takes about 1 h to train 200 epochs. To achieve better accuracy and lower loss, this task required a more complex structure than the first or second tasks, but the more complex structure meant that the model was prone to overfit. Thus, it was necessary to add more mechanisms for regularization, such as dropout, regularization, and batch normalization, as shown in Figure 12c. Figure 19 shows that the model quickly learned with an accuracy of about 0.6 in the first training before achieving an accuracy of 0.9 in the fine-tuning step. This indicates that a large number of pre-trained weights are needed to achieve a sufficient level when the dataset type is different.

**Figure 20:**
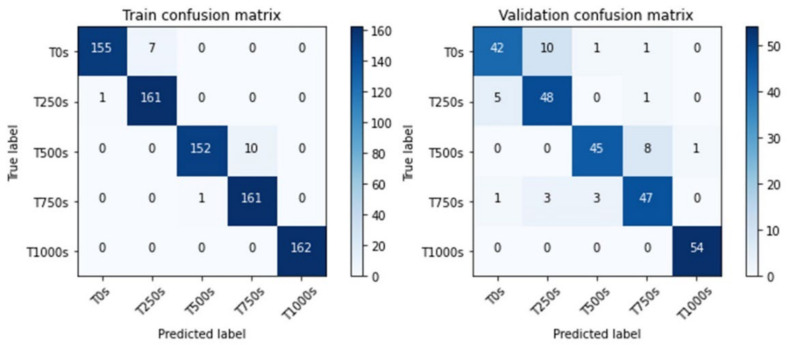
The confusion matrices of the model of the third task.

Figure 20 shows the confusion matrix of the training results. The model in Figure 20 has more errors when classifying the images of the first four categories and has a higher accuracy regarding the last category. This phenomenon results from the surface texture of the images. As shown in Figure 9, the surface of the abrasive belt after 1000 s of abrasion time was very fine, resulting in a much brighter image than the images of other categories. In addition to using the dataset with a coaxial white light source, the same model structure with the same training parameters was trained using the other two datasets with different light sources; the results are listed in Table 8. Similar to the results of the first task shown in Table 5, using the external coaxial white light yielded the best performance.

Both Table 5 and Table 8 show that among the three datasets using different light sources, the external coaxial white light had the best performance, followed by the high-angle ring light and then the external coaxial red light. This result differs from the conventional machine vision practice that states that the light source should be as close to the object’s appearance as possible. The reason might be that the data in the large dataset (ImageNet) used in the pretraining of the basic convolutional neural model Inception-ResNet-V2 were all in color, and the input of the model has also been designed for RGB trichromatic data. When using a single color light (in this case, red), the entire image is almost always in a red tone, meaning that the other two color lights (blue and green) are rather low in brightness, thus losing some features that help clarify the image. The reason for the better performance of the dataset with the external coaxial white light compared with the high-angle ring white light may be that the high-angle ring light did not equally illuminate different areas on the same workpiece. As shown in Figure 6, the brightness of the external coaxial white light image is more uniform than that of the high-angle annular white light image, making the features of the whole image more evenly detected. In these three experiments, each training only used a dataset with one kind of light source; thus, training with data crossing different light sources could be tested in the future. Sometimes, different light sources can give different perspectives about the surface information; datasets with different light sources could be input simultaneously, which may add some hidden information to the CNN models. In the future, estimation accuracy may be improved if the structure is fine-tuned and the stability of the grinding quality of the workpieces is enhanced or if the data from different light sources are input simultaneously.

Few papers have discussed the relationship between ground surface roughness and the degree of wear of abrasive belts. The current study briefly tested this: when other grinding parameters were fixed, the deterioration of the abrasive belt significantly affected the surface roughness. The abrasive belt is consumable, and it can grind the workpieces only for a certain time period. During the grinding process, the surface condition of the abrasive belt changes. Over time, large grits become small grits, and in this situation, the workpiece must be ground by a belt with a higher grit number. Therefore, given the workpiece and known abrasive belt, we could estimate the degree of wear of the abrasive belt by examining the pattern of the workpiece surface. More experiments can be conducted in the future to obtain a more accurate degradation curve of the abrasive belt. In addition, the current study only used Ra as a representative surface roughness parameter, while some studies have used other roughness parameters as indicators. The roughness parameters suitable for surfaces ground by various types of abrasive belts may be different; thus, more research is needed to find the most general roughness parameters.

The validation results of the three tasks confirm that a suitable CNN model with transfer learning and a small dataset could rapidly train a model capable of classifying various ground surfaces and estimating surface roughness. The benefits of this method are that it does not require a complex setup as the traditional roughness measurement apparatus does, and that it does not need additional complex image pre-processing procedures. The end-to-end training approach by deep learning can be quickly set up, trained, and deployed for different production lines.

This work confirms that the imaged-based, data-driven method can detect small pattern variations using only a small dataset without other pre-process and turning processes. The dataset can easily be sent for training after simple cropping and the standard augmentation step. The training process takes only 1–2 h.

## 4. Conclusions

In the current study, we have reported on an image-based inspection technique that only requires 750–1000 raw images and is capable of inspecting three kinds of tasks: classifying the grit number of the abrasive belt that grinds the workpiece, estimating the surface roughness of the ground workpiece, and classifying the degree of wear of the abrasive belts. The first and third tasks were classification problems, while the second was a regression problem. This study utilized a data-driven convolutional neural network with transferred learning of the model, which was trained using ImageNet. Thus, only a very small number of images taken specifically for the tasks was required, mainly for the first training and fine-tuning processes. The images of the ground workpieces were taken under three different light sources. They formed three datasets for performance comparison, including the external coaxial white light, high-angle ring light, and external coaxial red light. The experimental validation suggests that the model for the first and third tasks, classifying the grit number of the abrasive belt that grinds the workpiece and classifying the degree of wear of the abrasive belts, can achieve over 90% accuracy. As for the performance of the second task, about 70% of the evaluated samples can be estimated within a 25% error range, and the CoD between the estimated and actual values can reach 0.8. In short, the experimental results confirm that the textures of the images resulting from the geometrical variations under a light source can be utilized to represent the original geometrical characteristics, such as roughness. This would be difficult to detect by ordinary workers. The current study has also demonstrated that CNNs with transfer learning can distinguish surface textures with different types of surface roughness, thus providing a credible basis for subsequent applications of more complex and diverse neural networks on grinding surface image datasets.

The proposed methodology is quite useful and straightforward. However, we would also like to express that the results are exploratory and that the methodology can further be improved in many aspects, for example, expansion of the datasets, application on workpieces with curvy surfaces or materials, segmentation of image instances with convolutional layers, and so forth. We are in the process of tackling these issues. In addition, in the experiment, we have described the trend of the influence of the wear of the abrasive belt. However, it still needs more testing to acquire the curve of the relationship between the surface roughness (Ra) and abrasive time. In the future, experiments can further what has been done in the current paper, such as whether our technique can be applied to metal workpieces of different materials.

## Figures and Tables

**Table 1:** Workpieces and their corresponding manufacturing parameters.

Grit Number	Label	Grinding Process	Workpiece No.
#100	G100	X1	1
		X1	2
		X2	3
		X2	4
#150	G150	X1	5
		X1	6
		X2	7
		X2	8
#240	G240	X1	9
		X1	10
		X2	11
		X2	12
#400	G400	X1	13
		X1	14
		X2	15
		X2	16
#600	G600	X1	17
		X1	18
		X2	19
		X2	20

**Table 2:** Comparison of the grinding parameters in a factory setting and in this experiment.

Circumstance	Factory	Experiment
Normal force (N)	130	5
Grinding length per revolution (mm)	50	15
Contact area (mm × mm)	50 × 100	15 × 25
Normal force pressure (N/mm^2^)	0.026	0.1312
Length of the belt (mm)	3500	762
Linear velocity (mm/s)	16,338	16,000
Contact time per workpiece (s)	25	-
Workpieces before being worn out	150	-

**Table 3:** The accuracy of the various CNN models used as the base models.

Base Model	First Training		Fine Tuning		Dropout Ratio
	w/o Dropout	w/Dropout	w/o Dropout	w/Dropout	
ResNet V50	0.79	0.79	0.89	0.90	0.2
ResNet V152	0.83	0.81	0.86	0.88	0.3
Inception V3	0.76	0.67	0.88	0.87	0.5
InceptionResNet V2	0.75	0.72	0.88	0.92	0.5

**Table 4:** Accuracy of the models of the first task using the datasets of images illuminated by three different light sources.

	Datasets		
	External Coaxial Red Light	External Coaxial White Light	High-Angle Ring White Light
Mean	0.889	0.938	0.909
Standard Deviation	0.0150	0.0066	0.0158

**Table 5:** The training parameters of the model of the first task: classification of the grit number of the abrasive belt.

Training Parameters	Settings
Batch size	32
First training epochs	50
Fine-tuning epochs	150
Loss function	Categorical cross-entropy
Optimizer	Adam
First training learning rate	0.001
Fine-tuning learning rate	$1.00\times 10^{-5}$

**Table 6:** The training parameters of the model of the second task: Estimation of the surface roughness of the workpiece.

Training Parameters	Settings
Batch size	32
First training epochs	50
Fine-tuning epochs	300
Loss function	Mean square error
Optimizer	Adam
First training learning rate	0.001
Fine-tuning learning rate	1.00 × 10^−5^

**Table 7:** The training parameters of the model of the second task: Estimation of the degree of wear of the abrasive belt.

Training Parameters	Settings
Batch size	32
First training epochs	100
Fine-tuning epochs	100
Loss function	Categorical cross-entropy
Optimizer	Adam
First training learning rate	0.001
Fine-tuning learning rate	1.00 × 10^−5^

**Table 8:** Accuracy of the models of the third task using datasets of images illuminated by three different light sources.

	Datasets		
	External Coaxial Red Light	External Coaxial White Light	High-Angle Ring White Light
Mean	0.799	0.926	0.830
Standard Deviation	0.0251	0.0155	0.0136

## Data Availability

Not applicable.
